# Exploring Vegetarian and Omnivorous Approaches to Cardiovascular Risk and Body Composition

**DOI:** 10.3390/nu16132013

**Published:** 2024-06-25

**Authors:** Tatiana Fontes, Sofia Lopes, Regina Menezes, Marta Esgalhado, Luís Monteiro Rodrigues, Cíntia Ferreira-Pêgo

**Affiliations:** CBIOS—Universidade Lusófona’s Research Center for Biosciences and Health Technologies, Av. Campo Grande 376, 1749-024 Lisbon, Portugal

**Keywords:** body composition, lipid profile, cardiovascular risk, vegetarian diet, omnivorous diet

## Abstract

The role of nutrition in preventing non-communicable diseases has been widely studied in recent years, with indications that non-animal-based diets might improve body composition and therefore bring multiple health benefits. For all of these reasons, the main purpose was to compare body composition and metabolic status between vegetarian and omnivorous individuals and relate these values with cardiovascular risk. The present analysis included 176 participants (61 vegetarians and 115 omnivores). Body composition was assessed using a dual-energy X–ray absorptiometry, biochemical parameters obtained from capillary blood, and the 10-year cardiovascular risk (10RCVD) calculated by the QRISK3 score. No statistical differences were found between groups regarding body composition. Concerning metabolic markers, vegetarian individuals showed reduced values of total cholesterol, LDL cholesterol, and non–HDL cholesterol (*p* < 0.05). There were no differences in 10RCVD between groups. In both diets, moderate correlations between groups were found for cardiovascular risk and visceral adipose tissue. Our results suggest that the vegetarian regimen might be associated with better cardiometabolic biomarkers and better cardiovascular health, although controversial with the body composition trends observed. In conclusion, the results suggest that cardiovascular risk appears to be more influenced by body composition, mainly fat tissue, over dietary patterns itself.

## 1. Introduction

Non-communicable diseases are responsible for almost 90% of mortalities in Europe [1]. Obesity, defined as “*abnormal or excessive fat accumulation that may impair health*” [2], is a complex multifactorial disease universally graded by the body mass index (BMI) [1]. BMI, however, does not provide information regarding body fat distribution [3,4].

Several studies have reported an association between different fat compartments and cardiometabolic risk [3,5] thought to be related to an unhealthy distribution of fat or its excess [6]. Visceral adipose tissue (VAT) has been associated with increased cardiovascular risk and various health issues [4,7]. Some authors support the idea that VAT is an endocrine organ secreting adipocytokines, and strong evidence has shown that excess VAT leads to the development of dyslipidemia, hypertension, and insulin resistance [6]. Likewise, excess VAT has been linked to a higher prevalence of diabetes and high fasting glucose [8], hypertension [9], and dyslipidemia [10]. Subcutaneous adipose tissue (SAT), on the other hand, has been associated with a more protective role [8,9] and a reduced risk of diabetes and dyslipidemia [8].

Vegetarian diets, predominantly involving the consumption of plant-based foods while excluding the majority of, if not all, animal-based foods, have become increasingly popular in the last years [9,10]. Multiple health benefits have been reported when compared to omnivorous diets, and a properly planned, well-balanced vegetarian diet can provide all or nearly all required bodily nutrients [10]. Many plant-based foods are rich in flavonoids and carotenoids that might inhibit LDL cholesterol oxidation, leading to an increase in HDL cholesterol and, consequently, reducing concentrations of circulating total cholesterol and reducing the risk of atherosclerosis [11]. In turn, the high consumption of red meat and its derivatives seems to be related to an increase in mortality rates due to the high content of saturated fat present in these foods [12]. This high-fat content will contribute to an increase in LDL cholesterol and consequently result in the broader deposition of lipids in the vascular lumen, favoring sclerosis [11]. Some studies suggest that the increased consumption of vegetables and pulses, fruit, whole grains, and fish can lower all-cause mortality [12]. However, different authors argue that many of the associations between red meat and mortality may be influenced by other factors, including unhealthy lifestyle habits such as tobacco and alcohol consumption [12,13]. Vegetarian diets have been associated with higher weight loss, lower BMI, and, in some cases, a healthier distribution of body fat [10]. However, relationships between this regimen and body composition or adipose tissue distribution are not obvious [14]. Vegetarian diets have also been associated with lower values of systolic and diastolic blood pressure [15], triglycerides [16], and total and LDL cholesterol [17] compared to other omnivorous diets. People following vegetarian diets have been known to show better overall cardiovascular health metrics and lower healthy diet scores, likely due to the absence of fish consumption when compared to those following non-vegetarian diets [18]. However, these allegations are far from being accordant. 

The present study aimed to explore the cardiovascular impact of vegetarian and omnivorous dietary regimens by evaluating body composition and calculating a ten-year cardiovascular risk. 

## 2. Materials and Methods

### 2.1. Participants and Ethical Considerations

This cross-sectional observational study involved 176 previously selected participants (115 omnivorous and 61 vegetarian, 113 women and 63 men). Specific non-inclusion criteria included being pregnant or breastfeeding, being in the first week of the menstrual cycle, the regular use of medication that may evoke edema or dehydration, having an implanted pacemaker and/or any metal prosthesis, any previously diagnosed chronic non-communicable diseases, and practicing the respective dietary regimen for less than one year. All participants were residents of the Lisbon city district (Portugal) and were aged between 18 and 65 years old. The recruitment period took place between February 2022 and January 2023. The study was mainly disseminated through personal and institutional social networks. The sample size was calculated taking into consideration the last estimations for Portugal—76,000 people following a vegetarian dietary pattern. This represents a proportion of 0.9% of the total Portuguese population. This sample size calculation took into consideration a 95% confidence interval and an error margin of 5%.

All individuals agreed to participate in the study before data collection through informed written consent. Procedures respected all principles of good clinical practice adopted for human research studies, complying with current ethical standards for human research, following the Declaration of Helsinki [19] and respective amendments. The study was previously approved by the Ethics Committee of the School of Sciences and Health Technologies from Universidade Lusófona (EC.ECTS/P05.21). 

### 2.2. Data Collection

An interview preceded each assessment to (part 1) identify general participant characteristics of biological sex, age, family history of the disease, area of residence, place of residence (according to the division of the Nomenclature of Territorial Units for Statistical Purposes (NUT) II [20]), educational level, area of study, and monthly net household income. The International Physical Activity Questionnaire—Short Form was then applied (part 2) to obtain data on the level of physical activity for each participant [21]. Finally (part 3), a food frequency questionnaire (FFQ) validated for the Portuguese population was applied [22]. After integration into the study and based on the FFQ responses, participants were divided into two groups—following a vegetarian diet or following an omnivorous diet. The vegetarian group included all individuals who did not consume any type of animal products (meat, fish, or/and its derivatives). However, the consumption of dairy products, eggs, and honey was admitted. On the other hand, the omnivorous group included all individuals who consumed animal products (meat, fish, or/and its derivatives), with no type of “restriction” in this group. 

### 2.3. Measurement Outcomes

Body mass was measured by an electronic scale [0.1 kg (0.1–200 kg) accuracy]. Participants were wearing light clothes and no shoes. Height was a self-reported variable used to calculate BMI by the formula BMI = body mass (kg)/height (m^2^) [23]. Waist circumference (WC) was measured at the midpoint between the lower edge of the last palpable rib and the upper edge of the iliac crest. Finally, Dual-Energy X–ray Absorptiometry (DXA Lunar Prodigy Advance—General Electric Healthcare^®^; Chicago, IL, USA) was used to measure fat mass, fat-free mass, lean mass, bone mass, and VAT and SAT. Measurements were performed under twelve hours of fasting and exercise restriction within the twenty-four hours prior. The Appendicular Lean Mass Index (ALMI) was calculated using the formula [ALMI = (leg lean mass (kg) + arm lean mass (kg))/height (m^2^)] [24]. To identify excess fat mass, the NHAMES 1999–2014 cut-off for obesity diagnosis was used, considering excess fat mass values > 30% for men and values > 40% for women [25]. The lean mass deficit was identified using the EWGSOP2 cut-offs [26] for the ALMI, with a deficit being considered when women showed values < 5.5 kg/m^2^ and men values < 7.0 kg/m^2^. Although these cut-offs were created for older individuals [26], they have been recommended in the Global Leadership Initiative on Malnutrition—GLIM criteria for the adult population [27]. Health risk linked to waist circumference was classified according to WHO criteria, considering that the risk was increased when the values were ≥94 cm in men and ≥80 cm in women and substantially increased when the values were ≥102 cm in men and ≥88 cm in women [28].

Blood pressure and cardiac frequency were measured in the individual’s non-dominant arm with a digital sphygmomanometer. Three measurements were taken, discarding the first and averaging the other two. Reference values followed the European Society of Cardiology recommendations for systolic (SBP ≤ 120 mmHg) and diastolic blood pressure (DBP ≤ 80 mmHg) [29]. Mean Arterial Pressure (MAP), defined as the mean blood pressure throughout the cardiac cycle and a main determinant of perfusion and predictor of stroke risk [30] was also calculated using the formula [MAP = 1/3 × SBP + 2/3 × DBP]. A rapid testing device, the LINX DUO (A. Menarini Diagnostics^®^, Firenze, Italy), was used to assess metabolic markers in capillary blood, including glycated hemoglobin (Hemoglobin A1C), lipids (triglycerides, total cholesterol, and high-density lipoprotein [HDL]), and glucose. Low-density lipoprotein (LDL), very-low-density lipoprotein (VLDL), total cholesterol–HDL ratio, and cholesterol non–HDL were also quantified. The American Diabetes Association’s primary care cut-offs were used to classify excess hemoglobin A1c and fasting blood glucose [31]. Hemoglobin values ≤ of 5.7% and fasting glycemia values ≤ of 100 mg/dL were considered to be adequate [31]. Considering the recommendations of the European Society of Cardiology, evaluated parameters for triglycerides ≤ 150 mg/dL [32], total cholesterol ≤ 155 mg/dL [32], and LDL cholesterol ≤ 100 mg/dL [29] were regarded as normal. HDL cholesterol values >50 mg/dL for women and >40 mg/dL for men were considered acceptable [33]. The 10-year risk for cardiovascular disease (10RCVD) and the relative risk was calculated using the QRISK^®^3 score [34]. This algorithm uses risk equations, derived through Cox proportional hazards models, that are separate for men and women [34]. This algorithm has been released as open-source software under the GNU Lesser General Public Licence, version 3 [35].

### 2.4. Statistical Analysis

Statistical analysis was performed using the Statistical Package for Social Sciences (IBM SPSS) version 27.0 (SPPS Inc., Chicago, IL, USA). Data normality was assessed by the Kolmogorov–Smirnov test when *n* > 50 and the Shapiro–Wilk normality when *n* < 50 and parametric or non-parametric tests were chosen accordingly. Data were expressed as means (standard deviation, SD), median (interquartile range, IQR), and percentages (absolute values, *n*), according to the variable’s type. For comparison between categorical variables and scale variables, Student’s *t*-test and the Mann–Whitney U test were used. To compare two categorical variables, the Chi-squared, Fisher’s Exact, and Monte Carlo tests were used as appropriate. Pearson and Spearman’s correlations were also used. For better data visualization, scatter plots were created, allowing variable adjustment to the simple linear regression line. To evaluate the influence of confounding factors on body composition variables and biochemical parameters, multiple linear regression was used, creating two adjustment factor models. In Model 1, age, BMI, total energy value per weight unit, level of physical activity, smoking habits, academic qualifications, and dietary pattern were included as confounding factors. In Model 2, fat mass and VAT data were included along with all variables of Model 1. The models obtained obey Gauss–Markov conditions (residuals with zero mean, constant variance, and normal distribution). All statistical tests were two-tailed, and *p* ≤ 0.05 was adopted as the significance level.

## 3. Results

Details of the studied population are summarized in Table 1. Our study included 176 participants, of which 65.34% followed an omnivorous diet and 64.20% were women. Most participants were non-smokers (77.80%) and 38.60% had a BSc degree. The median participant age was 31 years old. Significant differences between groups were found regarding physical activity (*p* = 0.014) with the omnivorous group being associated with more moderate levels of physical activity (53.90% vs. 39.30%), and the vegetarian group showing more vigorous activities (39.90% vs. 19.10%). No other differences were found between the groups concerning body mass, height, BMI, or waist circumference.

Regarding macronutrient intake according to the dietary pattern (Table 2), we found that the OM population had a higher energy intake (OM: 2392.11 kcal vs. VG: 2066.40 kcal; *p* = 0.004) than the VG population. The OM group also presented a higher consumption of protein (OM: 18.00% vs. VG: 13.07%; *p* ≤ 0.001), saturated fat (OM: 9.43% vs. VG: 7.84%; *p* ≤ 0.001), and dietary cholesterol (OM: 349.25 mg vs. VG: 155.29 mg; *p* ≤ 0.001). On the other hand, the VG population presented a higher consumption of carbohydrates (VG: 50.20% vs. OM: 42.24%; *p* ≤ 0.001) and dietary fiber (VG: 35.32 g vs. OM: 30.85 g; *p* ≤ 0.027).

Regarding body composition (Table 3) no differences were found between both dietary patterns. However, our attention was drawn to some of the numbers observed in both groups, specifically, vegetarian women’s VAT (median: 205 cm^3^ vs. 173 cm^3^) and SAT (1053 cm^3^ vs. 898 cm^3^); vegetarian men’s fat mass (22.38% vs. 21.89%), VAT (median: 532 cm^3^ vs. 323 cm^3^), SAT (median: 947 cm^3^ vs. 875 cm^3^), and fat-free mass (77.61% vs. 78.11%) and ALMI (7.93 kg/m^2^ vs. 8.40 kg/m^2^). However, after adjusting for confounding factors, vegetarian men showed significantly lower values of lean mass (adjusted *p*-value = 0.013) and ALMI (adjusted *p*-value = 0.006).

Regarding cardiometabolic markers (Table 4), the omnivorous group showed statistically significant higher values of total cholesterol (OM: 183.83 mg/dL vs. VG: 159.77 mg/dL, *p* ≤ 0.001), LDL cholesterol (OM: 93 mg/dL, dL vs. VG: 83 mg/dL, *p* = 0.002) and non-HDL cholesterol (OM: 116 mg/dL vs. VG: 110 mg/dL, *p* ≤ 0.001) than the vegetarian group. These values remained statistically significant after adjusting for confounding variables.

Assessing the inadequacy of health outcomes (Table 5), omnivorous population depicted non-significant differences regarding fat mass (Men: 13.20% vs. 8%; Women: 19.50% vs. 11.10%) or lean mass (Men: 7.90% vs. 16.00%). For the metabolic markers, it was possible to verify that 50% of the men following an omnivorous diet showed elevated LDL cholesterol values and 31.60% reduced HDL cholesterol values, with these data being statistically significant between groups. These values remained statistically significant after adjusting for confounding variables. On the other hand, 47.20% of vegetarian women had statistically significant hemoglobin A1c, and 30.60% reduced HDL cholesterol. Women including animal products in their diet showed higher total cholesterol (79.20%, *p* = 0.042). When adjusting for confounding factors, it was also possible to verify that vegetarian women showed lower LDL cholesterol levels (adjusted *p*-value = 0.017).

Correlation analysis revealed that fat mass was positively correlated with triglycerides (r_s_ = 0.230, *p* = 0.013), total cholesterol (r = 0.393, *p* ≤ 0.001), LDL cholesterol (r_s_ = 0.291, *p* = 0.002), and 10RCVD (r_s_ = 0.259, *p* = 0.026) within the sample following an omnivorous diet (Table 6). VAT was positively correlated with all metabolic markers and cardiovascular risk parameters, as well as SAT, which was only not correlated with hemoglobin A1c and MAP. In the population practicing a vegetarian dietary pattern, fat mass correlated positively with hemoglobin A1c (r = 0.328, *p* = 0.010) and with LDL cholesterol (r = 0.292, *p* = 0.022). VAT correlated positively with 10RCVD (r_s_ = 0.583, *p* ≤ 0.001) and MAP (r_s_ = 0.510, *p* = 0.018), and SAT correlated negatively with HDL cholesterol (r_s_ = −0.261, *p* = 0.043) and positively with LDL cholesterol (r = 0.266, *p* = 0.038) and 10RCVD (r_s_ = 0.357, *p* = 0.010), in the same population.

**Figure 1:**
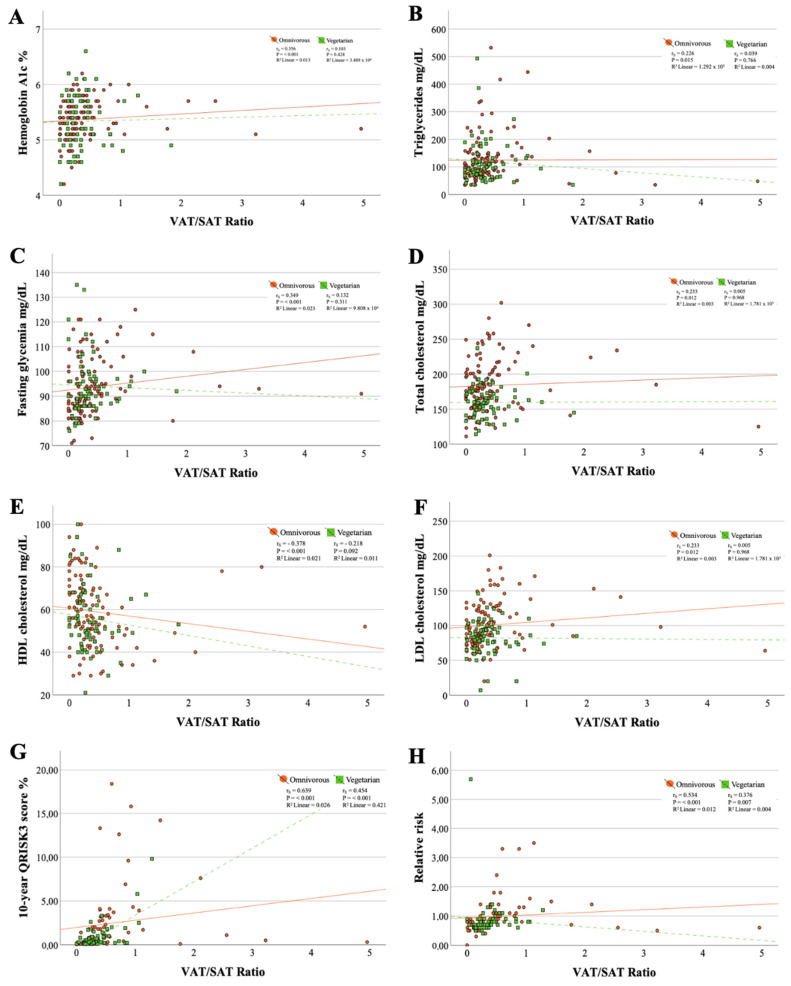
Scatter plots between metabolic markers and VAT/SAT ratio. (**A**) Correlation between hemoglobin A1c and VAT/SAT ratio; (**B**) Correlation between triglycerides and VAT/SAT ratio; (**C**) Correlation between fasting glycemia and VAT/SAT ratio; (**D**) Correlation between total cholesterol and VAT/SAT ratio; (**E**) Correlation between HDL cholesterol and VAT/SAT ratio; (**F**) Correlation between LDL cholesterol and VAT/SAT ratio; (**G**) Correlation between triglycerides 10-year QRISK3 score and VAT/SAT ratio; (**H**) Correlation between relative risk and VAT/SAT ratio.

Moderate correlations were found between the VAT/SAT ratio and the 10-year cardiovascular risk (omnivorous diet: r_s_ = 0.639, *p* ≤ 0.001 and vegetarian diet: r_s_ = 0.454, *p* ≤ 0.001) (Figure 1). Additionally, the goodness of fit of the simple linear regression line for these data revealed that for individuals following an omnivorous diet, the VAT/SAT ratio contributed only 2.6% (linear R^2^ = 0.026) to the variation of 10RCVD, while in participants practicing a vegetarian dietary pattern, this contribution was 42.1% (linear R^2^ = 0.421). All other correlations with the VAT/SAT ratio were weak or held in only one of the dietary patterns.

## 4. Discussion

The present study revealed no statistically significant differences between dietary groups and respective body composition. However, some specific aspects involving VAT and SAT detected in the vegetarian group have drawn our attention. Different interventions and case-control studies have associated vegetarian diets with lower values of body weight, BMI, and in some cases, total fat mass and VAT when compared with other, omnivorous diets [36,37]. Nevertheless, these results tend to be associated with intervention groups on a low-fat vegetarian diet and are compared to a control diet with no reduction in fat or other food groups, and sometimes there was an increase in food intake in these control groups [36,37]. In our study, we found that the total fat consumption of our sample of vegetarian individuals corresponded to 38% of the total daily energy value (TEV), of which approximately 8% corresponds to saturated fat. This high consumption of fat might explain the slightly elevated VAT and SAT values within the vegetarian group. Another study by Sofi et al. compared the effectiveness of a vegetarian diet vs. a Mediterranean diet (both with low energy value) and produced similar comments on body weight, BMI, and total fat mass, with no statistical differences between diets [38]. Shah et al. also found no statistically significant differences in BMI and waist circumference reduction when comparing a vegetarian diet with the diet recommended by the American Heart Association [39]. These studies demonstrate that when overall energy consumption is equivalent, no significant differences regarding body composition are shown [37,38]. In our study, similar fat consumption was also found in the omnivorous participants (39% of TEV), however, this group presented a higher moderate physical exercise practice, which likely may influence the VAT and SAT values [40].

In the analysis of body composition, it was also possible to verify that concerning ALMI, we found that men following a vegetarian dietary pattern had lower values (VG: 7.93 kg/m^2^ vs. OM: 8.40 kg/m^2^, *p* = 0.006) and a lower protein intake compared to those following a diet including animal-origin products (VG: 12.56% of TEV vs. OM: 18.61% of TEV, *p* = 0.002). Studies reported a statistically significant decrease in muscle mass and lean mass in men related to this plant-based diet allegedly associated with lower levels of leucine, an essential branched-chain amino acid responsible for muscle protein synthesis [40,41]. Leucine levels tend to be lower in vegetarians due to low or no intake of protein of high biological value [40,41]. 

The evaluation of metabolic markers indicated more accentuated differences between the dietary patterns. The vegetarian group presented significantly lower values of total cholesterol and LDL cholesterol compared to the omnivorous group. This is in line with results from other studies associating a vegetarian diet with lower levels of total cholesterol and LDL cholesterol [38,42]. Some studies suggested that low consumption of dietary cholesterol would involve a low rate of absorption and its conversion in the bloodstream [38,43], which would explain the low serum values in individuals following a vegetarian dietary regimen, as well as the higher intake of fiber here observed. However, the role of dietary cholesterol in reducing serum cholesterol levels is still a controversial issue with contradictory results [43]. There exists evidence suggesting that the consumption of saturated fat might be associated with increased LDL cholesterol and consequently increased cardiovascular risk, recommending a low consumption of saturated fat to control cholesterolemia [33]. In our study, we observed that the population following a vegetarian diet had a lower consumption of dietary cholesterol (155.29 mg vs. 349.25 mg, *p* ≤ 0.001) and saturated fat (7.84% of TEV vs. 9.47% of TEV, *p* ≤ 0.001) which could be a possible justification for the observed results. When results were analyzed considering the recommended cut-offs, we found differences between sexes that were not possible to verify in the population as a whole.

In our study, women following a vegetarian diet showed a higher percentage of hemoglobin A1c levels. Most studies on this issue suggest that vegetarian diets tend to be more beneficial for diabetes prevention control [43,44,45,46]. Consumption of dietary fiber, fruit, and vegetables has been related to lower levels of postprandial glucose and lipids [44,45]. However, most of these findings did not assess hemoglobin A1c levels or did not find reductions in its values [44,45]. A study conducted on Nepalese women found that vegetarians showed higher hemoglobin A1c levels, likely related to higher consumption of simple carbohydrates [47]. Dietary recommendations for individuals with diabetes and pre-diabetes emphasize the consumption of fruits, vegetables, whole grains, and low-energy protein [46]. The consumption of fats and simple carbohydrates (sugars) is also widely discussed in this pathology, and a daily energy consumption of more than 35% fats or 10% sugars is not recommended [46]. The female vegetarians in our study showed a high consumption of fat (40.08% of TEV, *p* = 0.989) and simple carbohydrates (17.29% of TEV, *p* = 0.247) which might contribute to explaining our results.

VAT and the VAT/SAT ratio showed moderate correlations with metabolic markers and/or cardiovascular risk. The omnivorous group showed non-significant correlations between VAT and all evaluated variables, while the vegetarian group showed non-significant correlations with MAP and cardiovascular risk. The other fat tissues showed weak correlations with these same variables. VAT volume has been associated with multiple cardiometabolic risk factors supporting the idea that VAT, besides contributing to general adiposity, also contributes to cardiometabolic risk [48]. This pathological potential of VAT has been associated with its ability to secrete pro-inflammatory and insulin-resistant adipokines. A study by Neeland et al. reported that obese individuals with high amounts of VAT also showed atherogenic dyslipidemia, hyperinsulinemia, and glucose intolerance [49]. The same profile was not present in obese individuals with low VAT values or in non-obese individuals, suggesting a relationship between higher VAT volumes and these cardiometabolic profiles [49], suggesting that VAT has a crucial impact on cardiovascular risk regardless of the dietary pattern. 

Our study provides a broad and rigorous view of the main players relating to diet, body composition, and cardiovascular risk, involving (a) deep analysis of known determinants such as age, weight, height, and BMI, complemented by body composition, metabolic markers, food intake, and lifestyles, while (b) exploring body composition through DXA, a precise and reliable technology serving our original purpose. Nevertheless, some limitations must be considered, such as (i) the size and heterogeneity of the sample; (ii) the cross-sectional design of the study not allowing a cause–effect relationship; (iii) the use of an unconventional method, using capillary blood, to evaluate the metabolic parameters, since some studies described the weak correlation between capillary and venous blood values [50,51]; and (iv) the use of self-reported height for the BMI calculation [52].

## 5. Conclusions

In conclusion, there were no differences in 10-year cardiovascular risk between these two dietary patterns. However, vegetarian participants displayed distinct macronutrient profiles showing a lower consumption of total energy, protein, saturated fat, and dietary cholesterol while exhibiting higher intakes of carbohydrates and dietary fiber compared to those on an omnivorous diet. These dietary variations may have contributed to the observed differences in metabolic markers, where vegetarians showed significantly lower levels of total, LDL, and non–HDL cholesterol. Dietary patterns by themselves do not seem to exert differential effects on specific aspects of body composition, BMI, or WC. Nevertheless, vegetarian men showed lower levels of lean mass and ALMI when adjusted for several confounding factors. Furthermore, correlation analysis revealed significant associations between fat mass, VAT, SAT, and cardiovascular risk, emphasizing the role of adipose tissue distribution in cardiometabolic health and risk. In this way, cardiovascular risk seems to be more influenced by body composition, fat mass distribution, and VAT and SAT levels, that is, by the diet quality rather than by the dietary regimen.

## Figures and Tables

**Table 1:** General characteristics of the study population, according to dietary pattern.

		All Population (*n* = 176)	Omnivorous (*n* = 115)	Vegetarian (*n* = 61)	*p*-Value
**Sex, % (*n*)**	Men	35.8 (63)	33.0 (38)	41.0 (25)	0.296 ^b^
	Women	64.2 (113)	67.0 (77)	59.0 (36)	
**Age, years**		31.0 (20.0)	30.0 (22.0)	33.0 (16.0)	0.185 ^a^
**Height, m**		1.7 (0.1)	1.7 (0.1)	1.7 (0.1)	0.169 ^a^
**Body mass, kg**		64.1 (21.3)	63.3 (21.4)	65.6 (20.0)	0.521 ^a^
**BMI, kg/m^2^**		22.5 (3.9)	22.4 (4.3)	22.7 (3.8)	0.756 ^a^
**Waist circumference, cm**	Men	83.0 (12.0)	83.0 (10.8)	85.0 (12.7)	0.855 ^a^
	Women	71.0 (13.7)	70.1 (14.0)	72.0 (11.9)	0.205 ^a^
**Academic qualifications, % (*n*)**	High school	34.7 (61)	33.9 (39)	36.1 (22)	0.895 ^b^
	BSc degree	38.6 (68)	38.3 (44)	39.3 (24)	
	MSc and PhD	26.7 (47)	27.8 (32)	24.6 (15)	
**Time following the dietary pattern, % (*n*)**	Between 1 to 5 years	14.2 (25)	0.0 (0)	41.0 (25)	<0.001 ^b^
	Between 5 to 10 years	14.2 (25)	0.0 (0)	41.0 (25)	
	More than 10 years	71.6 (126)	100.0 (115)	18.0 (11)	
**Smoking habits, % (*n*)**	Non-Smoking	77.8 (137)	77.4 (89)	78.7 (48)	0.656 ^b^
	Former smoker	11.4 (20)	10.4 (12)	13.1 (8)	
	Smoker	10.8 (19)	12.2 (14)	8.2 (5)	
**Levels physical activity, % (*n*)**	Low	25.0 (44)	27.0 (31)	21.3 (13)	0.014 ^b^
	Moderate	48.9 (86)	53.9 (62)	39.3 (24)	
	High	26.1 (46)	19.1 (22)	39.3 (24)	
**Family monthly income, % (*n*)**	<1000 €	10.2 (18)	7.0 (8)	16.4 (10)	0.127 ^b^
	1000–3000 €	59.1 (104)	60.0 (69)	57.4 (35)	
	>3000 €	30.7 (54)	33.0 (38)	26.2 (16)	

Data are expressed as a percentage (*n*) or median (IQR) for categorical or continuous variables, respectively. *p*-values for group comparisons between omnivorous and vegetarians were tested by ^a^ Mann–Whitney U Test or ^b^ Chi-squared, as appropriate. Abbreviations: BMI, Body Mass Index; BSc degree, bachelor’s degree; MSc and PhD, master’s and doctorate.

**Table 2:** Daily dietetic intake of the study population according to each sex and dietary pattern.

	All Population (n = 176)		*p*-Value	Men (n = 63)		*p*-Value	Women (n = 113)		*p*-Value
	Omnivorous (n = 115)	Vegetarian (n = 61)		Omnivorous (n = 38)	Vegetarian (n = 25)		Omnivorous (n = 77)	Vegetarian (n = 36)	
**Energy, kcal**	2392.11 (758.33)	2066.40 (602.13)	0.004 ^a^	2537.58 (1512.23)	1991.36 (572.76)	0.024 ^b^	2338.50 (706.31)	2119.11 (666.89)	0.120 ^a^
**Proteins, % TEV**	18.00 (5.46)	13.07 (6.59)	<0.001 ^b^	18.61 (6.64)	12.56 (6.72)	0.002 ^b^	17.76 (5.36)	13.88 (6.81)	<0.001 ^b^
**Carbohydrates, % TEV**	42.24 (11.97)	50.20 (18.09)	<0.001 ^b^	44.84 (11.73)	53.34 (10.20)	0.004 ^a^	43.35 (8.97)	47.57 (12.20)	0.041 ^a^
Complex, % TEV	15.62 (4.81)	17.91 (5.12)	0.004 ^a^	16.11 (6.64)	18.08 (6.16)	0.013 ^b^	14.81 (4.62)	18.35 (7.69)	0.077 ^b^
Sugars, % TEV	16.20 (6.16)	17.49 (8.33)	0.044 ^b^	15.49 (7.32)	18.35 (10.70)	0.095 ^b^	16.48 (5.69)	17.29 (6.48)	0.247 ^b^
**Total fat, % TEV**	39.72 (7.71)	38.38 (9.43)	0.311 ^a^	38.95 (7.96)	35.93 (8.57)	0.158 ^a^	40.10 (7.60)	40.08 (9.73)	0.989 ^a^
Saturated fat, % TEV	9.43 (2.47)	7.84 (2.76)	<0.001 ^a^	9.45 (3.93)	7.11 (4.88)	0.013 ^b^	9.66 (2.41)	8.11 (4.51)	0.004 ^b^
Monounsaturated fat, % TEV	20.44 (5.35)	20.84 (6.40)	0.665 ^a^	19.75 (4.82)	19.28 (5.63)	0.727 ^a^	20.78 (5.59)	21.91 (6.75)	0.351 ^a^
Polyunsaturated fat, % TEV	6.63 (1.60)	6.96 (2.43)	0.443 ^b^	6.70 (1.61)	6.37 (1.73)	0.653 ^b^	6.71 (1.20)	7.06 (1.67)	0.202 ^a^
**Cholesterol, mg**	349.25 (221.77)	155.29 (292.58)	<0.001 ^b^	350.03 (436.30)	45.00 (265.26)	<0.001 ^b^	349.03 (156.96)	229.06 (291.25)	<0.001 ^b^
**Dietary fiber, g**	30.85 (17.39)	35.32 (22.32)	0.027 ^b^	34.54 (20.49)	36.09 (21.92)	0.465 ^b^	29.61 (17.36)	34.06 (22.15)	0.039 ^b^
**Alcohol, g**	3.99 (5.97)	3.11 (4.66)	0.152 ^a^	5.07 (7.93)	2.12 (2.98)	0.019 ^a^	3.46 (4.70)	3.80 (5.46)	0.627 ^a^
Only consumers, g	3.18 (7.27)	2.58 (3.55)	0.285 ^b^	3.40 (7.47)	2.70 (2.86)	0.127 ^b^	2.71 (5.76)	2.52 (4.60)	0.829 ^b^
**Caffeine, mg**	41.35 (69.55)	34.83 (78.53)	0.443 ^b^	40.44 (79.53)	25.81 (79.38)	0.305 ^b^	41.35 (62.28)	41.11 (76.78)	0.961 ^b^
Only consumers, mg	41.35 (69.55)	34.89 (77.79)	0.551 ^b^	40.44 (79.53)	25.81 (79.38)	0.305 ^b^	41.35 (62.28)	46.24 (76.79)	0.770 ^b^

Data are expressed as a mean (SD) or median (IQR), as appropriate. *p*-values for group comparisons between omnivorous and vegetarians were tested by ^a^ Student’s *t*-test or ^b^ Mann–Whitney U Test, as applicable. Abbreviations: TEV, Total Energy Value.

**Table 3:** Body composition of the study population according to the dietary pattern sorted by sex.

	All Population (*n* = 176)		*p*-Value	Adjusted *p*-Value	Men (*n* = 63)		*p*-Value	Adjusted *p*-Value	Women (*n* = 113)		*p*-Value	Adjusted *p*-Value
	Omnivorous (*n* = 115)	Vegetarian (*n* = 61)			Omnivorous (*n* = 38)	Vegetarian (*n* = 25)			Omnivorous (*n* = 77)	Vegetarian (*n* = 36)		
**Fat mass, %**	29.23 (8.94)	28.38 (8.24)	0.534 ^a^	0.306	21.89 (6.80)	22.38 (6.28)	0.773 ^a^	0.096	32.85 (7.56)	32.52 (6.77)	0.827 ^a^	0.961
**VAT, cm^3^**	210.00 (583.00)	261.00 (482.00)	0.397 ^b^	0.945	323.00 (737.00)	532.00 (495.00)	0.550 ^b^	0.084	173.00 (377.00)	205.50 (323.00)	0.753 ^b^	0.180
**SAT, cm^3^**	893.00 (935.00)	1039.00 (842.00)	0.522 ^b^	0.074	875.50 (915.00)	947.00 (811.00)	0.911 ^b^	0.056	898.00 (983.00)	1053.00 (910.00)	0.373 ^b^	0.441
**VAT/SAT Ratio**	0.26 (0.31)	0.29 (0.29)	0.530 ^b^	0.183	0.40 (0.54)	0.46 (0.53)	0.347 ^b^	0.425	0.23 (0.26)	0.23 (0.20)	0.573 ^b^	0.199
**Fat-free mass, %**	70.77 (8.94)	71.63 (8.24)	0.535 ^a^	0.305	78.11 (6.81)	77.61 (6.29)	0.770 ^a^	0.181	67.14 (7.55)	67.47 (6.77)	0.827 ^a^	0.956
Bone mass, %	3.77 (0.54)	3.78 (0.58)	0.603 ^b^	0.573	3.87 (0.40)	3.98 (0.47)	0.300 ^a^	0.394	3.77 (0.60)	3.61 (0.52)	0.166 ^b^	0.191
Lean mass, %	67.01 (8.60)	67.84 (7.94)	0.532 ^a^	0.306	74.25 (6.60)	73.63 (5.98)	0.709 ^a^	0.013	63.44 (7.12)	63.83 (6.55)	0.785 ^a^	0.888
**ALMI, Kg/m^2^**	6.68 (2.20)	6.70 (1.71)	0.743 ^b^	0.306	8.40 (0.88)	7.93 (0.99)	0.055 ^a^	0.006	6.21 (0.89)	6.27 (0.72)	0.759 ^a^	0.650

Data are expressed as a mean (SD) or median (IQR), as appropriate. As appropriate, *p*-values for group comparisons were tested by ^a^ Student’s *t*-test or ^b^ Mann–Whitney U Test. Adjusted *p*-values were tested using multivariable linear regression, using model 1. Abbreviations: VAT, visceral adipose tissue; SAT, subcutaneous adipose tissue; ALMI, Appendicular Lean Mass Index.

**Table 4:** Cardiometabolic markers and cardiovascular disease risk of study population according to dietary pattern.

	All Population (*n* = 176)	Omnivorous (*n* = 115)	Vegetarian (*n* = 61)	*p*-Value	Adjusted *p*-Value
**Hemoglobin A1c, %**	5.40 (0.60)	5.40 (0.50)	5.30 (0.80)	0.613 ^b^	0.548
**Triglycerides, mg/dL**	102.00 (74.00)	107.00 (75.00)	99.00 (67.00)	0.715 ^b^	0.740
**Fasting glycemia, mg/dL**	91.00 (13.00)	91.00 (14.00)	92.00 (11.00)	0.682 ^b^	0.829
**Total cholesterol, mg/dL**	175.49 (35.48)	183.83 (38.47)	159.77 (21.79)	<0.001 ^a^	<0.001
**cHDL, mg/dL**	55.50 (20.00)	57.00 (22.00)	53.00 (19.00)	0.114 ^b^	0.222
**cLDL, mg/dL**	89.00 (36.00)	93.00 (46.00)	83.00 (28.00)	0.002 ^b^	<0.001
**cVLDL, mg/dL**	21.00 (15.00)	21.00 (15.00)	20.00 (13.00)	0.646 ^b^	0.510
**Total cholesterol/cHDL, mg/dL**	3.04 (1.16)	3.00 (1.29)	3.13 (0.89)	0.637 ^b^	0.159
**Non-HDL cholesterol, mg/dL**	112.00 (43.00)	116.00 (51.00)	106.00 (28.00)	<0.001 ^b^	<0.001
**SBP, mmHg**	110.00 (17.00)	110.00 (19.00)	110.00 (16.00)	0.667 ^b^	0.742
**DBP, mmHg**	74.00 (14.00)	74.00 (14.00)	73.00 (14.00)	0.311 ^b^	0.382
**MAP, mmHg**	86.33 (13.67)	87.00 (14.33)	84.67 (13.83)	0.356 ^b^	0.479
**Heart Rate, bpm**	67.00 (12.00)	67.00 (11.00)	67.00 (14.00)	0.494 ^b^	0.784
Cardiovascular disease risk *					
10-year QRISK3 score, %	0.70 (1.75)	0.90 (2.93)	0.40 (0.80)	0.077 ^b^	0.184
Relative risk	0.80 (0.40)	0.80 (0.43)	0.70 (0.30)	0.137 ^b^	0.990

Data are expressed as a mean (SD) or median (IQR), as appropriate. *p*-values for group comparisons between omnivorous and vegetarians were tested by ^a^ Student’s *t*-test or ^b^ Mann–Whitney U Test, as appropriate. Adjusted *p*-values were tested using multivariable linear regression, using model 2. * For this risk, only participants aged ≥ 25 years were included (Total population *n* = 125, Omnivorous *n* = 74, and Vegetarians *n* = 51). Abbreviations: cHDL, high-density lipoprotein cholesterol; cLDL, low-density lipoprotein cholesterol; MAP, mean arterial pressure; cVLDL, very-low-density lipoprotein cholesterol; DBP, diastolic blood pressure; SBP, systolic blood pressure.

**Table 5:** Inadequacy of body composition, anthropometrics, and metabolic markers of the study population, according to each sex and dietary pattern.

	Men (*n* = 63)		*p*-Value	Adjusted *p*-Value	Women (*n* = 113)		*p*-Value	Adjusted *p*-Value
	Omnivorous (*n* = 38)	Vegetarian (*n* = 25)			Omnivorous (*n* = 77)	Vegetarian (*n* = 36)		
**Body Composition, % (*n*)**								
Fat mass excess	13.20 (5)	8.00 (2)	0.693 ^a^	0.683 ^1^	19.50 (15)	11.10 (4)	0.268 ^b^	0.217 ^1^
Lean mass deficit	7.90 (3)	16.00 (4)	0.421 ^a^	0.404 ^1^	16.90 (13)	11.10 (4)	0.424 ^b^	0.685 ^1^
**Anthropometrics, % (*n*)**								
Increased WC	7.90 (3)	12.00 (3)	0.799 ^c^	0.193 ^1^	9.10 (7)	13.9 (5)	0.517 ^b^	0.970 ^1^
Substantially increased WC	2.60 (1)	0.00 (0)			18.2 (14)	11.1 (4)		
**Metabolic markers and vital signs % (*n*)**								
Elevated hemoglobin A1c	23.70 (9)	16.00 (4)	0.461 ^b^	0.637 ^2^	20.80 (16)	47.20 (17)	0.004 ^b^	0.009 ^2^
Elevated triglycerides	26.30 (10)	28.00 (7)	0.883 ^b^	0.624 ^2^	20.80 (16)	16.70 (6)	0.607 ^b^	0.745 ^2^
Elevated fasting glycemia	39.5 (15)	20.00 (5)	0.104 ^b^	0.344 ^2^	20.80 (16)	19.40 (7)	0.870 ^b^	0.874 ^2^
Elevated total cholesterol	68.40 (26)	52.00 (13)	0.189 ^b^	0.497 ^2^	79.20 (61)	61.10 (22)	0.042 ^b^	0.024 ^2^
Reduced cHDL	31.60 (12)	8.00 (2)	0.028 ^b^	0.036 ^2^	10.40 (8)	30.60 (11)	0.008 ^b^	0.002 ^2^
Elevated cLDL	50.00 (19)	20.00 (5)	0.016 ^b^	0.007 ^2^	40.30 (31)	22.20 (8)	0.060 ^b^	0.017 ^2^
Elevated SBP	23.70 (9)	20.00 (5)	0.731 ^b^	0.987 ^2^	7.80 (6)	0.00 (0)	0.174 ^a^	0.153 ^2^
Elevated DBP	26.30 (10)	20.00 (5)	0.565 ^b^	0.675 ^2^	14.30 (11)	8.30 (3)	0.543 ^a^	0.776 ^2^

Data are expressed as a percentage (*n*). *p*-values for group comparisons were tested by ^a^ Fisher’s Exact Test, ^b^ Chi-Square, or ^c^ Monte Carlo, as appropriate. Adjusted *p*-values were tested using multivariable linear regression, using ^1^ model 1 or ^2^ model 2 as appropriate. Abbreviations: WC, waist circumference; cHDL, high-density lipoprotein cholesterol; cLDL, low-density lipoprotein cholesterol; SBP, systolic blood pressure, DBP, diastolic blood pressure.

**Table 6:** Correlations between body composition and metabolic markers, vital signs, and cardiovascular disease risk according to dietary pattern.

	Fat Mass, %		VAT, cm^3^		SAT, cm^3^	
	Correlation	*p*-Value ^a,b^	Correlation	*p*-Value ^a^	Correlation	*p*-Value ^a,b^
**Omnivorous population**						
Hemoglobin A1c, %	0.058	0.537 ^a^	0.298	<0.001	0.078	0.410 ^a^
Triglycerides, mg/dL	0.230	0.013 ^a^	0.369	<0.001	0.349	<0.001 ^a^
Fasting glycemia, mg/dL	0.169	0.071 ^a^	0.414	<0.001	0.275	0.003 ^a^
Total cholesterol, mg/dL	0.393	<0.001 ^b^	0.317	<0.001	0.271	0.003 ^a^
HDL cholesterol, mg/dL	0.067	0.478 ^b^	−0.411	<0.001	−0.250	0.007 ^a^
LDL cholesterol, mg/dL	0.291	0.002 ^a^	0.429	<0.001	0.324	<0.001 ^a^
MAP, mmHg	0.027	0.772 ^a^	0.329	<0.001	0.172	0.066 ^a^
10-year QRISK3 score, %	0.259	0.026 ^a^	0.687	<0.001	0.380	<0.001 ^a^
Relative risk	0.193	0.100 ^a^	0.606	<0.001	0.395	<0.001 ^a^
**Vegetarian population**						
Hemoglobin A1c, %	0.328	0.010 ^b^	0.176	0.175	0.177	0.173 ^b^
Triglycerides, mg/dL	−0.029	0.825 ^a^	0.055	0.672	0.061	0.640 ^a^
Fasting glycemia, mg/dL	0.017	0.897 ^a^	0.169	0.193	0.061	0.641 ^a^
Total cholesterol, mg/dL	0.104	0.425 ^b^	0.120	0.357	0.079	0.543 ^b^
HDL cholesterol, mg/dL	−0.156	0.229 ^a^	−0.250	0.052	−0.261	0.043 ^a^
LDL cholesterol, mg/dL	0.292	0.022 ^b^	0.192	0.138	0.266	0.038 ^b^
MAP, mmHg	0.025	0.302 ^a^	0.510	0.018	0.194	0.133 ^a^
10-year QRISK3 score, %	0.203	0.154 ^a^	0.583	< 0.001	0.357	0.010 ^a^
Relative risk	0.136	0.341 ^a^	0.510	< 0.001	0.380	0.006 ^a^

^a^ Spearman and ^b^ Pearson correlations were performed, depending on the normal (or not) distribution of the sample. Abbreviations: VAT, visceral adipose tissue; SAT, subcutaneous adipose tissue.

## Data Availability

Data are unavailable due to privacy restrictions.
